# Tissue culture-free transformation of traditional Chinese medicinal plants with root suckering capability

**DOI:** 10.1093/hr/uhad290

**Published:** 2023-12-29

**Authors:** Jinghua Lu, Suhui Lu, Chunli Su, Shuai Deng, Mugui Wang, Huan Tang, Zhunian Wang, Guofu Li, Zhaobo Lang, Jian-Kang Zhu

**Affiliations:** Institute of Tropical Bioscience and Biotechnology/Sanya Research Institute, Chinese Academy of Tropical Agricultural Sciences, Haikou 570102, China; Institute of Crop Sciences/National Nanfan Research Institute, Chinese Academy of Agricultural Sciences, and Key Laboratory of Gene Editing Technologies, Ministry of Agriculture and Rural Affairs, Sanya 572024, China; Shandong Shunfeng Biotechnology Co. Ltd., Jinan 250000, China; Shandong Shunfeng Biotechnology Co. Ltd., Jinan 250000, China; Shandong Shunfeng Biotechnology Co. Ltd., Jinan 250000, China; Institute of Crop Sciences/National Nanfan Research Institute, Chinese Academy of Agricultural Sciences, and Key Laboratory of Gene Editing Technologies, Ministry of Agriculture and Rural Affairs, Sanya 572024, China; Tropical Crops Genetic Resources Institute, Chinese Academy of Tropical Agricultural Sciences, Haikou 570102, China; Sanya Research Institute & Tropical Crops Genetic Resources Institute, Chinese Academy of Tropical Agricultural Sciences, Sanya 572025, China; Shandong Shunfeng Biotechnology Co. Ltd., Jinan 250000, China; Institute of Advanced Biotechnology and School of Life Sciences, Southern University of Science and Technology, Shenzhen 518055, China; Institute of Crop Sciences/National Nanfan Research Institute, Chinese Academy of Agricultural Sciences, and Key Laboratory of Gene Editing Technologies, Ministry of Agriculture and Rural Affairs, Sanya 572024, China; Institute of Advanced Biotechnology and School of Life Sciences, Southern University of Science and Technology, Shenzhen 518055, China

Dear Editor,

For thousands of years, medicinal plants have played vital roles in treating and preventing diseases in the world. Enhancing the active ingredients of medicinal plants through modern biotechnological methods holds great promise for maximizing the therapeutic potential of these plants. However, genetic transformation has only been feasible for a very limited number of medicinal plant species, hampering the effort to improve these plants through genetic engineering or gene editing. Recently, we developed the cut-dip-budding (CDB) gene delivery system for plant transformation, which uses *Agrobacteria rhizogenes* K599 to induce transformed roots from explants and then obtain transformed buds from the transformed roots because of the root suckering ability [1]. Given that many medicinal plants also possess the characteristics of root suckering, we explored the applicability of CDB system in medicinal plants that are difficult or impossible to transform. Compared to the published CDB method, we optimized the infection procedure by suspending cultured *Agrobacteria* in MES buffer (pH 5.6), which contained 10 mM 2-(N-morpholino) ethanesulfonic acid (MES), 10 mM MgCl_2_, and 100 μM Acetosyringone (AS). We adapted the CDB method to transform medicinal plants as depicted (Fig. 1A).

**Figure 1:**
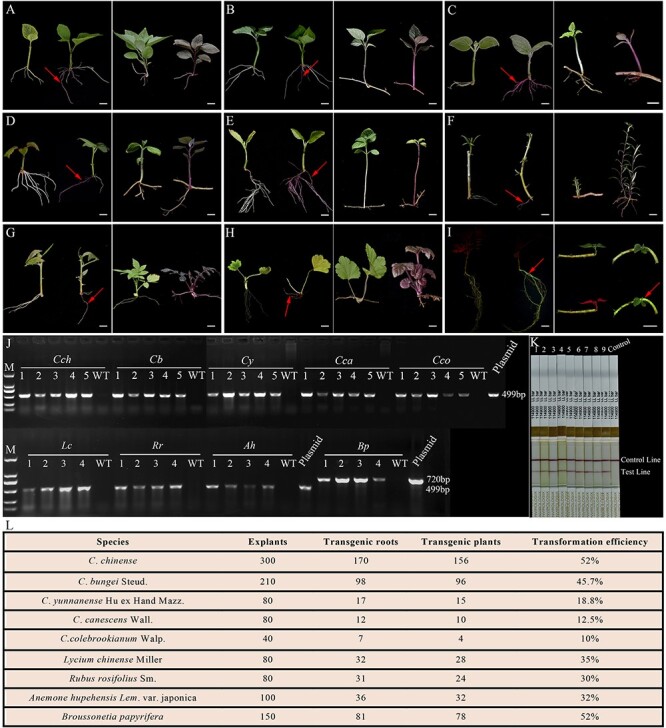
Transformation of medicinal plants using the CDB system. **A** CDB delivery system workflow. **B** RUBY-positive hairy roots and RUBY-positive *Clerodendrum chinense* (*Cch*) plant generated from the transformed roots. **C** RUBY-positive hairy roots and RUBY-positive *Clerodendrum bungei* (*Cb*) plant. **D** RUBY-positive hairy roots and RUBY-positive *Clerodendrum yunnanense* (*Cy*) plant. **E** RUBY-positive hairy roots and RUBY-positive *Clerodendrum canescens* (*Cca*) plant. **F** RUBY-positive hairy roots and RUBY-positive *Clerodendrum colebrookianum* (*Cco*) plant. **G** RUBY-positive hairy roots and RUBY-positive *Lycium chinense* (*Lc*) plant. **H** RUBY-positive hairy roots and RUBY-positive *Rubus rosifolius* (*Rr*) plant. **I** RUBY-positive hairy roots and RUBY-positive *Anemone hupehensis* (*Ah*) plant. **J** EGFP-positive hairy roots and EGFP-positive *B. papyrifera* (*Bp*) plant. **K** PCR results showing the presence of EGFP or RUBY reporter gene in positive shoots. **L** Bar protein detection results. **M** Statistics for the transformation efficiency of the nine medicinal plant species. In panels **A**–**I**, the plant on the left is an untransformed control. Red arrows point to a RUBY or EGFP-positive signal. Bar: 1 cm.

*Clerodendrum*, an important genus within the *Verbenaceae* family, has medicinal, ornamental, and research values [2–4]. Lack of a genetic transformation system has limited the improvement of these plant species. Considering that most *Clerodendrum* species have the root suckering ability, there is potential to establish a transformation system for these plants using the CDB approach. We used the RUBY reporter to test the applicability of the CDB gene delivery system in five different *Clerodendrum* species, including *Clerodendrum chinense*, *Clerodendrum bungei* Steud., *Clerodendrum yunnanense* Hu ex Hand Mazz., *Clerodendrum canescens* Wall., and *Clerodendrum colebrookianum* Walp. By infecting the young terminal buds or stems of these species with *A. rhizogenes* K599, hairy roots were formed approximately three weeks later. Some of these hairy roots were visually positive for RUBY (Fig. 1). Transgene positive RUBY red buds were generated from these RUBY roots after the roots were cultured in soil for 2–3 months. Finally, we obtained transgene positive shoots for *C. chinense* (Fig. 1B), *C. bungei* (Fig. 1C), *C. yunnanense* (Fig. 1D), *C. canescens* (Fig. 1E), and *C. colebrookianum* (Fig. 1F). These RUBY red shoots developed into normally growing plants that displayed the RUBY red color in all plant parts.

Having successfully applied the CDB gene delivery method to five species of *Clerodendrum* plants, we extended our efforts to transform four other medicinal plant species: *Lycium chinense* Miller, *Rubus rosifolius* Sm., *Anemone hupehensis* Lem. var. japonica and *Broussonetia papyrifera* Linnaeus. These selections were based on their root suckering ability, medicinal value and the difficulties in genetic transformation using traditional tissue culture-based methods [5–8]. Notably, genetic modification had not been reported for *A. hupehensis* and *R. rosifolius*. While transformation protocols exist for *L. chinense* and *B. papyrifera*, they are cumbersome and genotype dependent [9, 10]. We carried out the CDB protocol using RUBY as the reporter for the transformation of *L. chinense*, *R. rosifolius*, and *A. hupehensis*, and EGFP as the reporter for the transformation of *B. papyrifera.* Transgenic hairy roots formed approximately one month after infection with *Agrobacteria*. These transgene-positive hairy roots grew into healthy, normal-looking roots. Similar to the *Clerodendrum* species, transgene positive buds formed naturally on these roots without the need for root cutting (Fig. 1G–I). As *B. papyrifera* is a tall tree with a relatively long root suckering process, we expedited the generation of transgenic buds by cutting the transgene-positive roots into segments once they reached a certain thickness, and placing the segments on nutrient soil. Subsequently, transgenic buds emerged in 2–4 weeks (Fig. 1J).

All transgenic lines of the nine species of medicinal plants were confirmed to be transgene-positive through PCR and Bar (Bialaphos resistance/phosphinothricin acetyltransferase, a herbicide resistance gene) protein detection (Fig. 1K and L). To assess the efficiency of the CDB system, we conducted larger scale transformation experiments on these plants, yielding transformation efficiencies of 52%, 45.7%, 18.8%, 12.5%, 10%, 35%, 30%, 32%, and 52% for *C. chinense*, *C. bungei*, *C. yunnanense*, *C. canescens*, *C. colebrookianum*, *L. chinense*, *R. rosifolius*, *A. hupehensis* and *B. papyrifera*, respectively (Fig. 1M). These findings underscore the broad applicability and high efficiency of the CDB delivery system for the transformation of medicinal plants with root suckering capability.

In summary, we have successfully transformed nine species of traditional medicinal plants with root suckering capability using the CDB gene delivery system, providing compelling support to our hypothesis that this gene delivery system can be used to modify many plants with root suckering capability [1]. The application of the CDB system in the realm of medicinal plants carries substantial significance, as it opens up new avenues for enhancing these important species through transgenic or gene editing techniques. Future genetic improvements of medicinal plants have the potential to elevate the quality and consistency of herbal remedies, thereby increasing their efficacy for patients, and benefiting human health and well-being.

## Acknowledgements

This work was supported by Shandong Shunfeng Biotechnology Co. Ltd., Jinan, China, project of Sanya Yazhou Bay Science and Technology City (Grant No. SCKJ-JYRC-2023-72) and Hainan Yazhou Bay Seed Laboratory (Grant No. B22C10305).

## Author contributions

J.L., J.-K.Z. designed and supervised the research. J.L., S.L., C.S., S.D., and H.T. carried out the experiments. J.L., M.W., Z.W., Z.L., and J.-K.Z. discussed the data. J.L., G.L. Z.L., and J.-K.Z. wrote the paper.

## Data availability

All the data supporting the findings of this study are available in the paper, and the plasmid is available upon request.

## Conflict of interest statement

None declared.
